# Disclosure of HIV serostatus and condomless sex among men living with HIV/AIDS in Florida

**DOI:** 10.1371/journal.pone.0207838

**Published:** 2018-12-17

**Authors:** Christa L. Cook, Stephanie A. S. Staras, Zhi Zhou, Natalie Chichetto, Robert L. Cook

**Affiliations:** 1 Department of Nursing Systems, College of Nursing, University of Central Florida, Orlando, FL, United States of America; 2 Department of Health Outcomes and Biomedical Informatics, College of Medicine, University of Florida, Gainesville, FL, United States of America; 3 Department of Epidemiology, College of Medicine and College of Public Health and Health Professions, University of Florida, Gainesville, FL, United States of America; 4 Division of General Internal Medicine and Public Health, Vanderbilt University Medical Center, Nashville, TN, United States of America; 5 Department of Epidemiology, College of Medicine and College of Public Health and Health Professions, University of Florida, Gainesville, FL, United States of America; Agencia de Salut Publica de Barcelona, SPAIN

## Abstract

Despite campaigns to increase safer sex practices, there are people living with HIV/AIDS (PLWH) who do not disclose their HIV status to sexual partners and engage in condomless sex. The purpose of this research was to: 1) describe factors associated with disclosure of HIV status to sexual partners; and 2) determine if disclosure and/or receipt of prevention counseling are independently associated with condomless sex. We used the Florida Medical Monitoring Project to analyze data from 376 HIV positive men with more than one sexual partner. Results indicated that 55% consistently disclosed their HIV status to sexual partners, 30% inconsistently disclosed, 15% did not disclose, and 48% reported any condomless sex. The odds of having condomless sex was 3.3 (CI = 1.5, 7.3) times greater in men who disclosed to all partners. Results suggest that men who disclose are also those who are more likely to have condomless sex. More research is needed to better understand the complex nature of disclosure and sexual risk behaviors and how disclosure impacts sexual risk.

## Introduction

Despite worldwide campaigns to increase safer sex practices, some people living with HIV/AIDS (PLWH) fail to disclose their HIV status and engage in condomless sex with partners who are HIV negative, potentially leading to increased rates of disease transmission. In recent years, 24 states have made failure to disclose a positive HIV status to a sexual partner a criminal offense.[1] There is a general agreement these laws should be re-evaluated since there insufficient evidence that they are effective, they may lead to increased stigma, and they potentially interfere with prevention efforts such as partner notification.[1] In Florida specifically, it is a third-degree felony with up to 5 years in prison and/or a $5000 fine if someone living with HIV does not disclose their status to sexual partners.[2] For the potential partner, knowledge of the person’s HIV status could play a critical role in making informed decisions about sexual practices, such as the use of condoms or pre-exposure prophylaxis [PrEP] that can drastically reduce the rate of transmission in serodiscordant relationships.[3] Furthermore, mathematical modeling indicates a 38% to 61% reduced risk of HIV transmission when PLWH disclose their status to potential sexual partners.[4] Even though disclosure brings about the potential to decrease the risk of disease transmission through increasing protective measures, the social consequences are less straightforward, and researchers have reported inconsistent findings on the factors associated with disclosure, or the effects of disclosure on rates of risky sexual behavior and disease transmission.[5–18]

Many PLWH state that they fail to disclose their positive HIV status because they fear rejection and stigmatization by others.[19] This fear may explain the wide variation in the proportion of PLWH who disclose their HIV status: disclosure to all partners ranges from 23%-93%, disclosure to some partners and not others ranges from 31%-38%, and disclosure to no partners ranges from 22%-47%.[4–7,11,13,15,20–22] However, other, less studied factors that may be associated with disclosure include HIV clinical status (time living with HIV, CD4+ T-cell count, HIV viral load), sociodemographic factors (race, poverty, education), substance abuse and mental health symptoms, HIV knowledge, and counseling programs.[6,16,18,20,22–31]

Significantly, several predictors of disclosure are modifiable and can be targeted by public health researchers who seek to develop risk prevention interventions.

Most studies of disclosure have focused on populations recruited from clinical trials. Surveillance data may be more representative of the general population. Thus, in this study, we use supplementary surveillance data, which can provide a representative population-based sample, to understand the contextual factors that may be associated with the relationship between disclosure and condomless sex. Therefore, the purpose of this research is to: 1) describe factors, including sociodemographic characteristics, clinical and health status factors, sexual risk behaviors, and receipt of prevention counseling, that are associated with disclosure of HIV status to sexual partners; and 2) determine if disclosure and/or receipt of prevention counseling are independently associated with condomless sex among sexually active men living with HIV.

## Methods

### Study sample and data collection

The Centers for Disease Control and Prevention fund and coordinate the Medical Monitoring Project (MMP), a national supplemental surveillance project for PLWH. The MMP uses a multi-stage sampling design with the goal of selecting a sample with equal probabilities of selection to the entire HIV/AIDS population receiving care in the United States.[32] MMP participants recruited for surveillance are diagnosed with HIV, 18 years old or older, and enrolled in care at a sampled facility. For this study, we used 5-years of data (2009–2013) from the MMP completed in Florida, which ranks second in the nation in newly diagnosed HIV cases and first in age-adjusted mortality from HIV/AIDS.[33,34] We limited the analytic sample to men who were sexually active in the past year and had 2 or more sexual partners because the US epidemic has disproportionately impacted men,[35] and previous research has shown that men with multiple sexual partners are less likely to disclose their HIV status.[20,36] Furthermore, including persons with only one partner could bias our analyses because they would have a different number of possible “disclosure” category options than those with multiple partners, as noted below. Participants who were newly diagnosed in the past year were excluded. Of the 2078 MMP participants, 791 (38%) were sexually active in the past 12 months, and 430 (21%) had multiple sexual partners. After excluding participants identifying as women or transgender and participants who were diagnosed in the same year as the data were collected, data from a total of 376 men were analyzed. This analysis of the Florida MMP data received approval from the University of Florida Institutional Review Board prior to study commencement.

### Measures

#### Condomless sex

The primary dependent variable was the report of having anal or vaginal sex without a condom with at least one sexual partner in the past 12 months.

#### Disclosure of HIV status to sexual partners

Participants were asked “During the past 12 months, did you discuss your HIV status with either your causal and/or your main partners”. The disclosure of HIV status was organized into three categories: disclose to none (participants answered no for *all* casual and main partners), disclose to some (participants answered yes to *some*, *but not all* casual or main partners), or disclose to all sexual partners (participants answered yes to *all* casual and main partners).

#### HIV prevention counseling

Participants were asked if they had participated in a one-on-one conversation with an outreach worker, counselor, prevention program worker, doctor, nurse, or other health care worker about ways to protect themselves or others from transmitting HIV during the 12 months prior to the survey.

#### Covariates

Covariates were chosen based on previous associations with HIV infections and sexual-risk taking identified in the literature and described in the introduction. Socio-demographic variables were self-reported and included age, race/ethnicity, education and poverty level. Poverty level was calculated by dividing the participants’ reported income by the number of dependents and classified using Department of Health and Human Services Poverty Guidelines, based on year of interview.[37] Sexual behavior was grouped into two categories: 1) men who have sex with men (MSM), including those men who had sex with both men and women (MSMW), or 2) men who have sex with women exclusively (MSW). We chose to combine MSM and MSMW due to small numbers of MSMW in this sample. Years of HIV infection was defined as the time since the first positive test for HIV. HIV viral load results in the past 12 months were determined by medical record abstraction, and durable viral suppression was defined as having a suppressed viral load (≤200 copies/mL) at each time of testing in the last 12 months.[38] A Patient Health Questionnaire depression scale (PHQ-8) score of ≥10 was used to define current depression.[39] Other covariates included substance use before or during sex in the past 12 months, (“during the past 12 months, did you use any alcohol, non-injection, or injection drugs before or during sex”) and total number of sexual partners in the previous year (“in the past 12 months, with how many different men/women have you had oral, vaginal, or anal sex”).

### Analysis

We used weighted bivariate analysis to describe the factors associated with disclosure of HIV status to sexual partners, using specific recommendations from the MMP analytical guidelines for subsamples [40]. We chose the modified Rao-Scott Chi-square test, which is appropriate when analyzing categorical variables from complex survey data, such as the MMP, and statistical significance was defined at the p-value level of ≤ .05. Weighted multivariable logistic regression analysis was conducted to assess the association between disclosure of HIV status and having condomless sex. Covariates were included in the multivariable model if the covariates were significantly associated with having condomless sex in the bivariate analysis (p≤.05). No collinearity was found between covariates. Crude and adjusted odds ratios with 95% confidence limits were estimated. All data analyses were performed in SAS version 9.4 (SAS Institute, Carry, NC, USA).[41] Although all analyses were conducted using the weights assigned by the Centers for Disease Control [40], we report the actual number of participants in the results.

## Results

Characteristics of sexually active men living with HIV who had more than one sexual partner are summarized in Table 1. Participants in the sample (n = 376) were generally older than 35 years of age (84%), having sex with men (83%), lived above the poverty level (71%), had been diagnosed with HIV for 3 or more years (86%), were receiving ART (93%), and had a consistently-suppressed HIV viral load (71%). Although 48% of participants engaged in condomless sex, 52% of participants reported that they used a condom during all sexual encounters.

**Table 1:** Factors associated with disclosure of HIV status in 376 men with multiple sexual partners living with HIV.

	Total	Disclosure to none	Disclosure to some	Disclosure to all	p-value
	n (%)	56 (15%)	112 (30%)	206 (55%)	
**Age**					
18–34 yrs.	61 (16)	10 (16.2)	24 (41.1)	27 (42.8)	0.53
35–44 yrs.	94 (25)	15 (15.3)	28 (30.9)	51 (53.8)	
45–54 yrs.	157 (42)	19 (10.8)	39 (30.9)	97 (52.4)	
> = 55 yrs.	64 (17)	12 (16.9)	21 (30.7)	31 (52.4)	
**Race and ethnicity**					
White	170 (46)	14 (8.2)	58 (36.7)	97 (55.1)	0.03
Black	110 (30)	24 (20.4)	38 (34.4)	48 (45.1)	
Hispanic or Latino	87 (24)	15 (14.3)	14 (23.6)	57 (62.1)	
**Education**					
<High School	40 (11)	10 (24.4)	7 (19.2)	23 (56.4)	0.27
High School diploma	82 (22)	12 (13.4)	25 (35.2)	45 (51.4)	
>High School	254 (67)	34 (12.3)	80 (33.7)	138 (53.9)	
**Poverty level**					
Above	256 (71)	36 (13.4)	81 (34.4)	137 (52.2)	0.48
At or below	106 (29)	17 (14.5)	27 (27.7)	62 (57.8)	
**Sexual behavior**					
MSM	311 (83)	35 (10.9)	102 (35.5)	172 (53.6)	< 0.01
MSW	65 (17)	21 (31.1)	10 (15.4)	34 (53.5)	
**Depression**					
No depression	309 (83)	48 (14.1)	96 (34.2)	163 (51.7)	0.16
Major Depression	65 (17)	8 (13.0)	15 (23.0)	42 (64.0)	
**Years with HIV**					
< = 3 years	53 (14)	7 (10.9)	21 (46.8)	25 (42.3)	0.13
>3 years	323 (86)	49 (14.4)	91 (30.0)	181 (55.6)	
**Currently taking ART**					
No	28 (7)	7 (23.5)	8 (30.8)	13 (45.7)	0.49
Yes	348 (93)	49 (14.4)	104 (32.7)	193 (54.2)	
**HIV viral load**					
All undetectable	247 (71)	37 (13.8)	78 (33.3)	130 (52.9)	0.96
Detectable	101 (29)	14 (12.5)	28 (33.3)	59 (54.3)	
**Prevention counseling**					
No	144 (39)	25 (15.8)	40 (27.0)	78 (57.2)	0.51
Yes	230 (61)	31 (12.8)	71 (35.6)	127 (51.6)	
**Number of partners**					
2–3	192 (51)	31 (14.2)	29 (13.8)	132 (71.9)	< 0.01
4–5	73 (20)	12 (18.5)	24 (32.4)	37 (49.1)	
>5	109 (29)	13 (10.3)	59 (60.4)	37 (29.4)	
**Alcohol/drug before sex**					
No	137 (37)	26 (17.7)	32 (26.5)	79 (55.8)	0.12
Yes	237 (63)	29 (11.4)	80 (36.2)	126 (52.4)	

### Bivariate analysis

#### Factors associated with disclosure

Of the sexually active men living with HIV, 55% disclosed their HIV status to all partners, 30% disclosed their HIV status to some of their partners and 15% did not disclose their HIV status to any partners (Table 1). As shown in Table 1, four factors were significantly associated with disclosure: race/ethnicity, sexual behavior, number of partners, and use of a condom. In terms of race and ethnicity, on average, the proportion of participants who disclosed to none of their partners was greater in white participants than black participants. In terms of sexual behavior, a greater proportion of MSW disclosed to none of their partners (Table 1). In terms of number of sexual partners, there was an inverse relationship between the proportion of men who disclosed to all partners and the total number of sexual partners. Finally, persons who had condomless sex tended to be more likely to disclose to some or all of their partners, whereas those who always used condoms (no condomless sex) were more likely to disclose to none of their sexual partners. Factors not significantly associated with disclosure included age, education, poverty level, depression, years with HIV, taking ART, HIV viral load, and receipt of prevention counseling.

#### Factors associated with condomless sex

Condomless sex (Table 2) was reported by 48% of the sample. Six variables were associated with having higher than expected condomless sex: white race and ethnicity, greater than high school education, above the poverty level, sex with men, a greater number of partners, and using alcohol or drugs before sex.

**Table 2:** Factors associated with having condomless sex in 376 men with multiple partners in previous year.

	*No condomless sex* *186 (52%)*	*Having condomless sex* *169 (48%)*	*p-value*
**Age**			
18–34 yrs.	26 (43.5)	31 (56.5)	0.24
35–44 yrs.	45 (49.4)	45 (50.6)	
45–54 yrs.	75 (47.9)	70 (52.1)	
> = 55 yrs.	40 (60.3)	23 (39.7)	
**Race and ethnicity**			
White	66 (39.3)	99 (60.7)	< 0.01
Black	69 (62.8)	33 (37.2)	
Hispanic or Latino	44 (51.9)	35 (48.1)	
**Education**			
<High School	28 (71.8)	10 (28.2)	< 0.01
High School diploma	48 (55.8)	30 (44.2)	
>High School	110 (44.2)	129 (55.8)	
**Poverty level**			
Above	113 (44.0)	128 (56.0)	< 0.01
At or below	65 (64.9)	36 (35.1)	
**Sexual behavior**			
MSM	130 (43.1)	163 (56.9)	< 0.01
MSW	56 (88.7)	6 (11.3)	
**Depression**			
No depression	156 (49.8)	137 (50.2)	0.88
Major Depression	30 (51.0)	30 (49.0)	
**Years with HIV**			
< = 3 years	25 (46.9)	25 (53.1)	0.67
>3 years	161 (50.2)	144 (49.8)	
**Currently taking ART**			
No	15 (55.6)	12 (44.4)	0.55
Yes	171 (49.3)	157 (50.7)	
**HIV viral load**			
All undetectable	121 (48.6)	112 (51.4)	0.20
Detectable	53 (56.0)	42 (44.0)	
**Prevention counseling**			
No	76 (51.6)	60 (48.4)	0.59
Yes	110 (48.9)	108 (51.1)	
**Number of partners**			
2–3	117 (61.3)	62 (38.7)	< 0.01
4–5	33 (48.7)	35 (51.3)	
>5	36 (34.2)	72 (65.8)	
**Alcohol/drug before sex**			
No	85 (64.9)	44 (35.1)	< 0.01
Yes	99 (40.9)	125 (59.1)	
**Disclosure of HIV status**			
To none	42 (79.1)	10 (20.9)	<0.01
To some	49 (44.4)	60 (55.6)	
To all	94 (45.5)	98 (54.5)	

Note: there are only 355 men included in this table because 21 participants did not report sexual behavior.

### Multivariable logistic regression

#### Disclosure and condomless sex

In multivariable analysis, when compared to men who did not disclose to any partners, the odds of having condomless sex was 3.3 (95% confidence interval = 1.5, 7.3) times greater in men who disclosed to all partners and, while not statistically significant, 1.5 (95% confidence interval = 0.7, 3.2) times greater in men who disclosed their HIV status to some of their partners. Other factors that were strongly associated with condomless sex included sex with women exclusively (aOR 7.16, 95% confidence interval = 3.25, 15.75), alcohol or drug use before sex (aOR 2.30, 95% confidence interval = 1.32, 4.02), and more than 5 sexual partners in the past year (aOR 3.24, 95% confidence interval = 1.53, 6.86) (Table 3).

**Table 3:** Factors associated with condomless sex in 376 men with HIV and multiple sex partners in the past year: multivariable analysis.

	Adjusted Odds Ratio	95% Wald Confidence Limits	
**Disclosure of HIV status**			
Disclose to none (ref)			
Disclose to some	1.48	0.68	3.19
Disclose to all	3.33	1.51	7.33
**Race and ethnicity**			
White (ref)			
Black	0.85	0.53	1.37
Hispanic or Latino	0.74	0.41	1.34
**Poverty level**			
Above (ref)			
At or below	1.29	0.47	3.53
**Education**			
<High School (ref)			
High School diploma	1.10	0.39	3.08
>High School	1.61	0.53	4.84
**Sexual behavior**			
MSM (ref)			
MSW	7.16	3.25	15.75
**Alcohol/drug before sex**			
No (ref)			
Yes	2.30	1.32	4.02
**Number of partners**			
2–3 (ref)			
4–5	1.84	0.85	3.95
>5	3.24	1.53	6.86

## Discussion

In this population-based sample of HIV positive men who had multiple sexual partners in the past year, we found that about half of the participants consistently disclosed their HIV status and almost half engaged in condomless sex. We also found men who disclosed their HIV status to all sexual partners were 3.3 times more likely to engage in condomless sex than men who did not disclose their HIV status to any partners. Compared to prior studies in men that separated disclosure into three categories (disclosure to none, some, or all), in our study the proportion of men who disclosed their HIV status to all of their sexual partners was higher (55% vs. 23–39%) and the proportion of men who disclosed their HIV status to none of their partners was lower (15% vs. 30–33%).[6,22] Sampling differences are one possible reason for differences in our results and previous studies, some of which were done exclusively in urban areas and others in which disclosure laws differed.[1,6,22] Another possible reason for differences from other samples is that our analysis only included men with multiple sexual partners.

In this analysis, having an undetectable viral load was not associated with disclosure of HIV status or having condomless sex, potentially indicating that participants were unaware of the reduced transmission of HIV with undetectable viral loads. The MMP data was collected between 2009–2013, before and immediately following the publication of results from the HTN 052 in 2011 demonstrating treatment as prevention [42], Some previous studies have indicated that those who are on ART or who are virally suppressed are less likely to disclose their HIV status to partners, possibly because they believe they are not capable of disease transmission.[6,43,44] Future research is needed in this area to assess if the population based sample vs. the clinical trial sample can account for these differences and whether there were changes in disclosure after treatment as prevention was implemented.

It is noteworthy that in this sample, prevention counseling was not significantly associated with rates of disclosure or condomless sex. Thus, our findings support those of other studies, which have found similar results. For example, in an analysis of national 2009 MMP data, 61% of those receiving risk reduction counseling still engaged in condomless sex.[45] Other research has also found no long-term differences in disclosure or condom use among those receiving a risk reduction counseling intervention.[46] The failure to a find an association between risk reduction counseling and disclosure of HIV status may be due to the variety of counseling approaches. Notably, successful interventions promoting disclosure involved repeated sessions of prevention counseling targeted specifically towards disclosure of HIV status.[36] Furthermore, recent research suggests non-disclosure and engagement in condomless sex may be associated with decreased self-efficacy to disclose.[43] Because approximately 76% of providers report providing risk reduction counseling, [47] it is imperative that public health researchers evaluate the use of these interventions.

Our results suggest that men who disclose are also those who are more likely to have condomless sex. Our findings of increased disclosure associated with decreased condom use are not consistent with most other studies, perhaps because they included different samples (e.g., clinical trial participants or population based), differences in how the outcomes were measured (dichotomous or 3-levels), recruitment areas (urban/rural), and whether the research was conducted in areas that have disclosure laws.[1,4–16,21–23]. It also could be that the association between disclosure and condom use differs by partner type (e.g. main or casual), but we were not able to clearly link disclosure and condom use by individual partner type in these analyses. An increased understanding of the role of seroadaptation (i.e. serosorting and seropositioning), a harm reduction approach of choosing partners and sexual positions based on HIV status [48] is needed to broaden the interpretation of these results and evaluate accurate counseling strategies.

There are several limitations to this study. First, we only examined the association of disclosure of HIV status and use of a condom with sex from the perspective of MMP participants and thus we were unaware of their partners’ HIV status or use of PrEP.[18,21] Second, the sample was recruited from those in care in the State of Florida and decisions regarding disclosure and condomless sex may differ for those who are not receiving care or who reside in other areas of the United States. With Florida criminal laws for non-disclosure, it is also possible MMP participants underreported non-disclosure. Finally, condom use is usually lower with primary partners and relationship status was not included as a covariate. However, a strength of this study is the use of the MMP data that yields representative population estimates of PLWH who are receiving care.

This study highlights the need for further research on the effect of prevention counseling on rates of disclosure and condomless sex. Specifically, we need to understand whether or not disclosure leads to more condomless sex, or how disclosure may influence discussions about prevention. Furthermore, as pre-exposure prophylaxis (PrEP) becomes more of an HIV prevention option, [3] we need to understand how disclosure may impact discussions about PrEP. Criminalization of HIV non-disclosure complicates both the individual decision and the relationship between the provider and client, and clinical guidelines emphasize the legal aspects of disclosure more than the prevention aspects.[18,49] Qualitative methods, especially grounded theory, may help us further understand the processes and contextual factors that influence the relationship between disclosure and condomless sex.
